# Alternative Techniques for Porous Microparticle Production: Electrospraying, Microfluidics, and Supercritical CO_2_

**DOI:** 10.1007/s11095-025-03923-2

**Published:** 2025-09-11

**Authors:** Simon Pöttgen, Christian Wischke

**Affiliations:** Martin-Luther-University Halle-Wittenberg, Institute of Pharmacy, Kurt-Mothes-Str. 3, 06120 Halle, Germany

**Keywords:** Electrospraying, Microfluidics, Microparticles, Porosity, Supercritical fluids

## Abstract

Microparticles have been established as injectable drug carriers designed to enable a long-term release of the encapsulated active pharmaceutical ingredients (API). To regulate this release, the diffusion barrier provided by the matrix material – typically hydrolytically degradable polyesters – must be controlled through precise levels of matrix porosity. This mini-review presents processing methods that are alternatives to the most common batch emulsification techniques for the manufacturing of porous polymer particles. A focus is placed on mechanistically describing the particle and pore formation in droplet-based microfluidics, electrospraying, and by supercritical fluids, critically discussing their opportunities and challenges. Ultimately, this review assesses the potential of these techniques in advancing the engineering of porous polymeric carrier systems in the light of scale-up and continuous production.

**Graphical Abstract:**

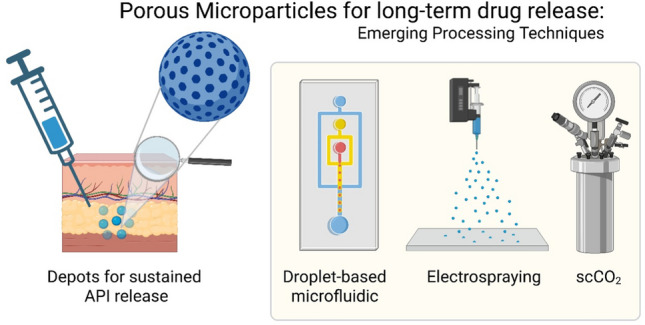

## Introduction

Microparticulate drug carriers are an established concept in the development of long-acting drug products. This technology continues to be highly interesting for creating new prospective treatment options and clinical applications [1–3]. Since its implementation, the class of microparticulate drug carrier products is dominated by hydrolytically degradable polyesters as matrix materials such as polylactide (PLA), poly(lactide-co-glycolide) (PLGA), or poly(ε-caprolactone) (PCL). While other alternative materials may also be relevant for drug delivery [4–6], it is worthwhile to improve, e.g., PLGA-based particulate drug carriers to reach optimal release rates [7–9].

From the perspective of formulation development, the characteristics of each fabrication method in combination with the tunable range of formulation parameters/process conditions can result in different morphological features of microparticles. In particular, the modification of their ultrastructure, namely the particle porosity by suitable fabrication strategies can have a major impact on their performance as drug carriers. These structures can be critical quality attributes (CQAs) given their potential contribution in regulating drug release kinetics [10–12].

The field of porous particle preparation from polyester materials is dominated by batch emulsification processes [10, 13], with spray drying techniques also playing a relevant role [14, 15]. However, advances in the field of fabrication methods led to less common techniques like droplet-based microfluidics, electrospraying, or the treatment with supercritical fluids, which gained increasing attention in the recent decade [16, 17]. In particular, such alternative techniques can be of interest, specifically if they enable continuous manufacturing, which could contribute to cost reduction, higher product safety, and easier quality controls [18, 19]. Although parenteral controlled release products are not used at quantities like peroral dosage forms, which are currently focused on in pharmaceutical factories for transition to continuous manufacturing, evaluating the applicability of continuous methods can also be relevant for particulate carriers for various reasons. For instance, product quality may be improved if the production method can be combined with a continuous monitoring (and control) of, e.g., particle diameters as an important CQA. Furthermore, overlying processes during particle formation (e.g., solvent/antisolvent flux, polymer precipitation, osmotic processes) and pore creation (e.g., by porogen leakage or osmotic processes) motivate the evaluation of such type of strategies, which may timely separate the particle templating and the creation/fine-tuning of porosity in independent process steps.

Interestingly, despite the relevance of the degree of porosity and of pore structures for the release function of particulate carriers, this topic is not systematically addressed in research studies and only partially covered in the standard analysis of particulate drug carriers.

In this review, we will focus on the fabrication of porous drug carrier systems – mainly made of synthetic polyesters like PLGA, PLA, and PCL – by using alternative manufacturing methods like electrospraying, droplet-based microfluidics, and supercritical fluid treatment and give a short overview on methods characterizing particle porosity. We will briefly describe the principles of these methods, provide examples of their application to generate porous particles, and critically evaluate the challenges and opportunities of the methods in terms of productivity and reproducibility compared to conventional methods. This review should also contribute to drawing more attention to the inclusion of quantitative analysis of particle porosity in the portfolio of standard characterizations reported in scientific studies on particulate drug carriers made from hydrophobic polymers.

## Principles of Pore Creation and Pore Characteristics

Pores are products of phase separation processes, i.e., defects in the bulk polymer matrix. These defects are typically formed on the basis of statistical processes (which means by chance), e.g., when water enters the (organic) polymer phase in emulsion processes.

Pores in polymeric microparticles can have high structural diversity, for instance, in terms of diameters, aspect ratios (width/length), or tortuosity. Pores can be open, i.e., directly accessible from the particle surface, or closed, meaning that the void can only be accessed by diffusion processes through the bulk. These differences in ultrastructure can have a significant effect on the general occurrence and kinetics of exchange processes. In particular, open pores can enable the entrance of the dispersion medium into a particle, at least if the material is sufficiently wettable, thereby supporting mass transport of substances into and out of the particle bulk through their inner surfaces via direct contact with the dispersion medium.

Pores in polymer microparticles are only seldom of homogeneous sizes, with few exceptions. Therefore, one should be aware that reported pore sizes typically would be a mean value that is representative of a certain pore size distribution. However, there is no established criterion or threshold to characterize this size distribution in the field of injectable polymeric drug carriers. Evidently, along with other structural parameters as well as drug solubility and diffusivity in the (potentially hydrated) polymer matrix, pore size distributions will affect mass transportation. In principle, two particle batches with similar average pore sizes, of which one exhibits a mixture of large and small pores leading to the same numeric value of average pore sizes, may behave differently in terms of mass transfer, as relevant for drug release, polymer degradation rates, as well as mechanical stability of particles during handling. The coefficient of variation (CV), which is occasionally used to describe the width of particle/droplet size distribution for those techniques leading to uniform particle sizes like droplet-based microfluidics [20], would be an appropriate measure also for pore size distributions. As a threshold, a CV > 40% is here suggested to quantitatively characterize broad pore size distributions. Still, as CV values of pore size distributions are typically not reported, any statement on broad or narrow size distributions in this review will be based on a qualitative assessment in the respective references or a visual assessment of published images by the authors.

### Porogens

**Fig. 1:**
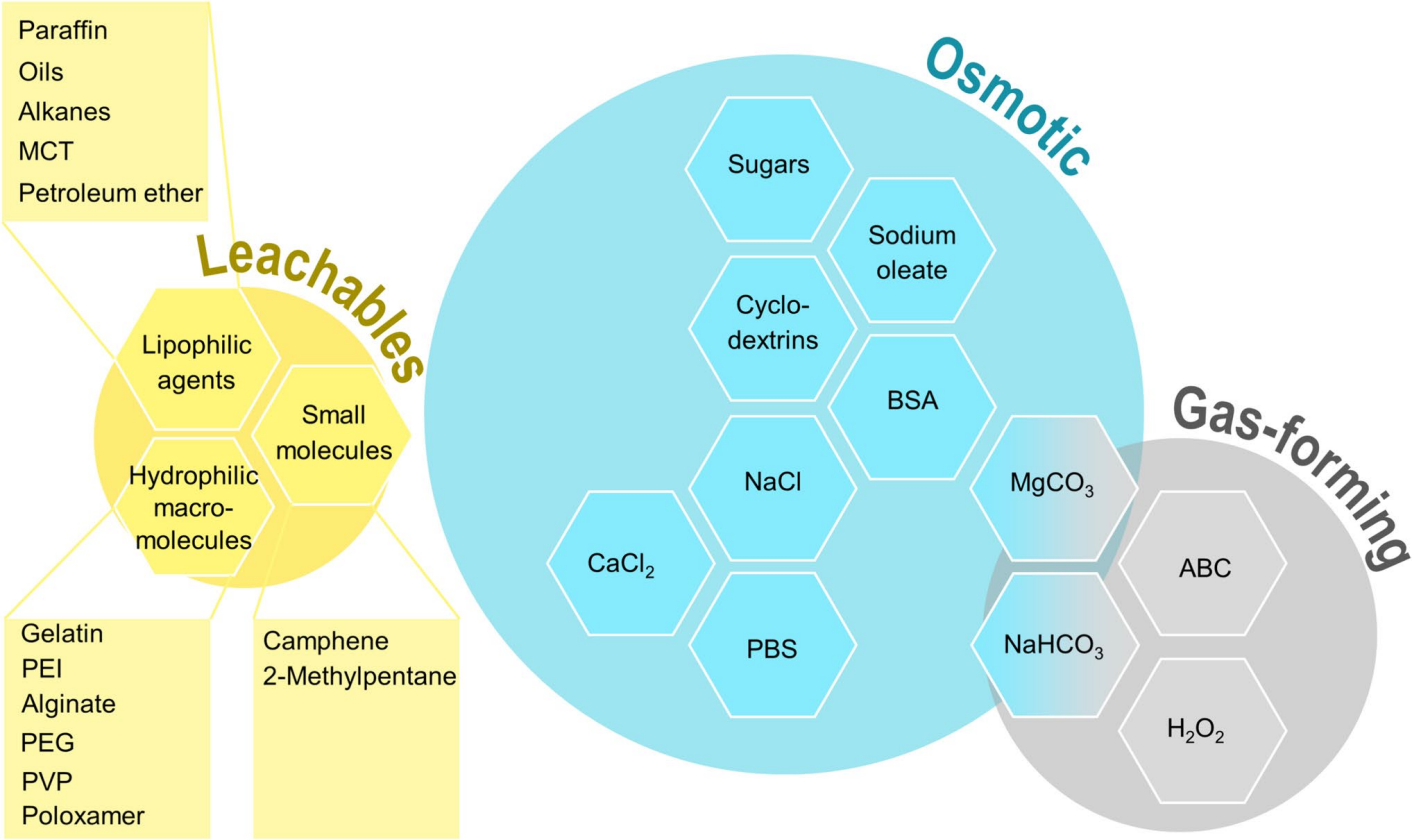
Scheme of different classes of porogens (blue: osmotic; grey: gas-forming; yellow: leachables). Examples of commonly used substances within each class are shown in honeycomb cells, while the size of the background colour reflects their employment in literature.

Pore formation is, in most cases, supported by the use of porogens. These pore-forming substances typically support phase separation processes in nascent particles. Porogens can subsequently, at least to a major extent, be removed from the particle matrix. For instance, some porogens can be extracted by certain solvents after particle solidification or can transition to a gaseous state (evaporation), leaving behind voids in the respective material [21]. According to their mechanisms of action, porogens can be classified into different groups, like gas-forming, osmotic, or leaching/extractable agents [10]. Examples of substances used as porogens and their relative contribution to the scientific literature are summarized in Fig. 1.

In principle, most porogens are compatible with different matrix materials from which porous particles should be produced. Porogens can also be compatible with several fabrication methods, often allowing to enhance the overall porosity and to tailor the pore sizes of polymeric scaffolds, including microparticles, in a controlled fashion [22–24]. However, some porogens, for instance those being based on osmosis, will only work with particle production techniques that at some point involve phase boundaries to an aqueous phase. It should not be forgotten that there can be limitations in the degree of porosity of polymer microparticles and thus in the use of porogens. For instance, overly high porosity in combination with, e.g., brittleness of very thin polymer struts in the scaffold can result in instability and collapse/fracturing of the particles.

#### Osmotic Agents

Osmotic active substances are the most prominent and widely used class of pore-forming agents. Various substances, for example different salts and sugars (NaCl, CaCl_2_, PBS buffer, sucrose) that are low in costs, can act as osmotic porogens either by being dissolved in an encapsulated aqueous phase or by being suspended in the organic polymer phase during emulsion-based particle preparation.

The mechanisms of pore formation by osmotic agents is the following: During particle preparation by emulsion techniques, the organic solvent of the polymer solution is gradually extracted to the external medium. At the same time, osmotic agents promote the influx of water from the aqueous continuous phase into the organic polymer phase. This solvent exchange may interfere in various ways with the particle solidification, depending on the respective selected combinations of material and solvent properties, phase volumes, etc., which may promote a faster or slower polymer precipitation at phase boundaries. Though in most cases a major shrinkage of solidifying particles is observed after some time [25, 26] (most protocols keep stirring for 2-12 h), a swelling of nascent particles can be expected immediately after droplet formation, if the matrix material can absorb water. Furthermore, the incorporation of water in the semi-solidified polymer particles may cause pore formation/pore opening at the particle surfaces during the final drying step, which is typically conducted by freeze drying [10].

Osmotically active agents may contribute to pore formation also via mixed effects. For instance, they may operate through osmosis plus gas formation (carbonates, hydrogen carbonates) or through osmosis plus leaching when being flushed out by water as very small particles from the nascent (larger) polymer microparticles [27, 28]. As we will discuss in Sect."Opportunities and Challenges of Alternative Methods to Prepare Porous Particles", where manufacturing methods are described, osmotic porogens have been used in microfluidic particle production. Although mass transport mediated via osmotic effects can be sensitive to process scale, the use of osmotically active porogens also appears suited for continuous processing in principle. This is particularly true where scale-up does not proceed in the classical fashion through larger vessels, but uses the numbering-up approach as applicable for microfluidics.

#### Gas-forming Agents

Those agents are conceptually elegant as they should be completely and autonomously removed from the product. Examples of regularly employed substances are ammonium bicarbonate, other bicarbonate salts (sodium and magnesium bicarbonate), and hydrogen peroxide [29, 30] (Fig. 1).

The underlying principle of gas-forming agents is either their physical transition to a gaseous state or their participation in a chemical reaction, generating gas as one of the reaction products. Obviously, this transition must be inducible at the process conditions of particle preparation, which may involve shear stress, heat development, shifts of pH-values, or hydration of the porogen. In this way, volatile gases like carbon dioxide, ammonia (e.g. in case of ammonium bicarbonate), oxygen, or hydrogen (e.g. from hydrogen peroxide) may be formed. The individual gas molecules accumulate in small bubbles in the polymer matrix and template pores in the material while evaporating.

Besides the use of gas-forming porogens in emulsion processes, the expansion of pressurised gas from its supercritical state is another principle of pore creation via gas bubbles. This principle is implemented in processes employing scCO_2_, as will be discussed in more detail in Sect."Opportunities and Challenges of Alternative Methods to Prepare Porous Particles".

Interestingly, porogens of this class have been used in different studies in a remarkably broad concentration range covering, e.g., 1–15% (w/v) in the w_1_ respective phases [31, 32]. When using relatively aggressive substances, such as oxidizing agents or strong bases, potential detrimental effects on the stability of the matrix polymer or the payload should be tested. In terms of continuous manufacturing, examples of producing porous particles with gas-forming porogens in microfluidic systems are also available (Sect."Opportunities and Challenges of Alternative Methods to Prepare Porous Particles"). However, at larger scales and with higher porogen concentrations, the amount of gas released will be high, which can be problematic in closed channels. Upscaling should therefore be carefully evaluated and tested to avoid disrupting the manufacturing process. For instance, in the worst cases, high gas concentrations may cause inhomogeneities in the batch product. Furthermore, air bubbles captured in closed systems and pressure fluctuation in microfluidic systems due to moving air pockets may interfere with production processes.

#### Leaching Agents

In this class of porogens, numerous materials are reported ranging from lipophilic oils to polymeric substances [10]. Depending on the used polymer matrix, extractable porogens may either be miscible with this material or may be phase separated as solids, fluids, or gels.

The fundamental principle of using extractable porogens is based on solvents, which dissolve the porogen, but not the matrix material of the particles. Through this principle, at some point in the production process, porogens initially embedded in matrix material are removed, leaving pores behind [33, 34]. However, the extraction of these substances sometimes involves harsh extraction procedures, such as organic solvents for lipophilic porogens. More hydrophilic macromolecular porogens like gelatin can typically be removed within a few hours using a warm water bath [10, 34, 35].

There are different strategies for implementing extraction/washing steps in continuous processes, some of which are further discussed in Sect."Opportunities and Challenges of Alternative Methods to Prepare Porous Particles". For example, particles incorporating extractable porogens may be exposed to solvents in extraction baths with a certain particle residence time as defined, e.g. by sedimentation speeds. The prepared particles may also be introduced in special counterflow channels that can be coupled, e.g., to microfluidic systems, in which the extractant is brought in contact with the particle dispersion. Further useful techniques to realize a separation of solids (particles) from fluids (continuous phase; extraction medium) may be based on centrifugation, as preparative continuous flow centrifugation is well established for other use cases in the pharmaceutical sector and life sciences [36–38]. Accordingly, these methods may be evaluated to extract leachable agents and to provide a control of the duration of incubation in washing solutions.

### Self-assembly of Materials

Self-assembly is a term used to describe supramolecular behaviour of substances that can organise themselves into certain geometric shapes through intermolecular interactions [39]. Prominent examples of these processes can be seen by the formation of micelles or colloids [40]. In general, self-assembly processes are often observed for surfactants and for block copolymers (BCP), which contain both hydrophilic and lipophilic block segments [41].

The concept of cubosomes – a term often used in the field of lipid-based particles with a defined porous structure [42, 43] – has recently been expanded to the field of polymers. Polymer cubosomes as highly porous particles can be constructed via self-assembly from block copolymers, such as those with an asymmetric diblock structure and a dominating hydrophobic nature. These polymers form cubic-shaped assemblies during micro-phase separation with water-filled voids between the more lipophilic segments. They further transform into small solid microparticles (often less than 5 µm) or nanoparticles with numerous homogeneously sized pores, typically in the size range of mesopores. The asymmetric molecular structure of the block copolymers must involve a larger lipophilic segment (often hydrophobic fraction > 90%) to mediate the assembly [44].

The formation of polymer cubosomes often involves solvent exchange processes, such as titration of the polymer solution with a non-solvent (nanoprecipitation) or solvent-diffusion-evaporation methods, while other methods also exist [45]. For instance, the predominately hydrophobic block copolymer can be dissolved in a suitable hydrophobic solvent (e.g. dioxane/DMF mixtures), followed by the addition of a good solvent for the hydrophilic segments (e.g. water) to induce nanoprecipitation [46]. The produced pore system is composed of two simultaneously existing networks of channels, one being open to the surface and a second one laying in between the other network and being not accessible from the surface, at least initially.

As this class of particles has only recently been developed, the current state of investigation is still in the process of fundamentally understanding the conditions of controlled self-assembly, often using polymers that are more relevant for technical settings rather than the pharmaceutical sector. However, block copolymers based on PLA and other degradable materials with potential applications as pharmaceutical matrix systems have been reported, suggesting their general applicability in the field of pharmaceutics and continuous drug release [47]. Importantly, their highly defined structures with high porosity and very narrow pore size distributions make them interesting as standardisable carrier systems, which, hypothetically, also includes the possibility of post-modifications like coatings to tailor release rates.

## Opportunities and Challenges of Alternative Methods to Prepare Porous Particles

### Electrospraying

Electrospraying allows the dispersion of a liquid into fine droplets by electrohydrodynamic atomization. The setup consists of a high voltage power supply, a syringe pump, a conductive nozzle and a grounded collector. For particle production, polymer solutions are pumped through the nozzle and, in the applied electric field, form a fluid cone at the tip of the nozzle. Charged droplets are ejected from this cone due to interfacial instabilities. The droplets subsequently shrink and solidify by solvent evaporation during their passage in the gas phase towards the collector [17, 48]. Electrospraying can produce particles with a narrow size distribution, homogenous surface characteristics, and tunable particle properties like porosity depending on the fine-tuning of process conditions [49, 50]. Various polymers such as PCL [50–53], PLGA [54–57], PLA [58, 59] PMMA [60], Eudragit^®^ [61], or biomacromolecules like gelatin and chitosan [62, 63] have been processed to porous microparticles by electrospraying.

In order to obtain porous particles by this technique, typically low polymer concentrations/low fluid viscosities are required. Relevant formulation and process parameters, which can affect the particle morphology, include the solvent type [64–67], the polymer or drug concentration [52, 55, 57, 68], the presence of low concentrations of pore forming agents such as ammonium bicarbonate [54, 59], the flow rates for feeding the polymer solution to the nozzle [62, 69], the applied voltages [62, 70], the collector distance [69, 71], and in many cases environmental conditions such as temperature and humidity [60, 72]. Numerous studies have evaluated changes in different process parameters (e.g., solvent types, polymer concentrations, flow rates, voltages, etc.) and screened the resulting particle properties to set parameter values for the desired particle morphology [52, 54, 58, 62, 64, 65, 68, 69]. Particularly the type of solvents is a crucial factor for the particle morphology and porosity. Mixtures of solvents and non-solvents (for the given polymeric material) were applied at different ratios to increase the porosity of PCL [53] and PMMA [60] particles. For instance, dichloromethane (DCM), a solvent for PCL, was blended with ethanol, a non-solvent for PCL, resulting in a shift of the pore size distribution toward larger average diameters as the ethanol content increased. Interestingly, at the highest ethanol concentration, a sharp decrease in pore size was observed, which was interpreted as the expulsion of ethanol from the nascent particles, thereby limiting its contribution to pore formation [53]. For the preparation of porous PMMA particles by electrospraying, again DCM was used as a solvent in combination with hexanol, ethanol, and propanediol as non-solvents, tested at varying humidity conditions [60]. These methods are believed to operate through a non-solvent induced phase separation (NIPS), wherein non-solvent droplets form during particle hardening that eventually cluster to a network of connected pores. While the use of hexanol resulted in spherical porous particles, ethanol and propanediol led to less spherical and partially hollow particles, suggesting that the more rapid phase separation induced by these more hydrophilic alcohols caused surface perturbations in the nascent particles [60]. With increasing humidity of the air environment in the spraying chamber, the pore diameters increased for samples containing hexanol in the solvent mixture.

**Fig. 2:**
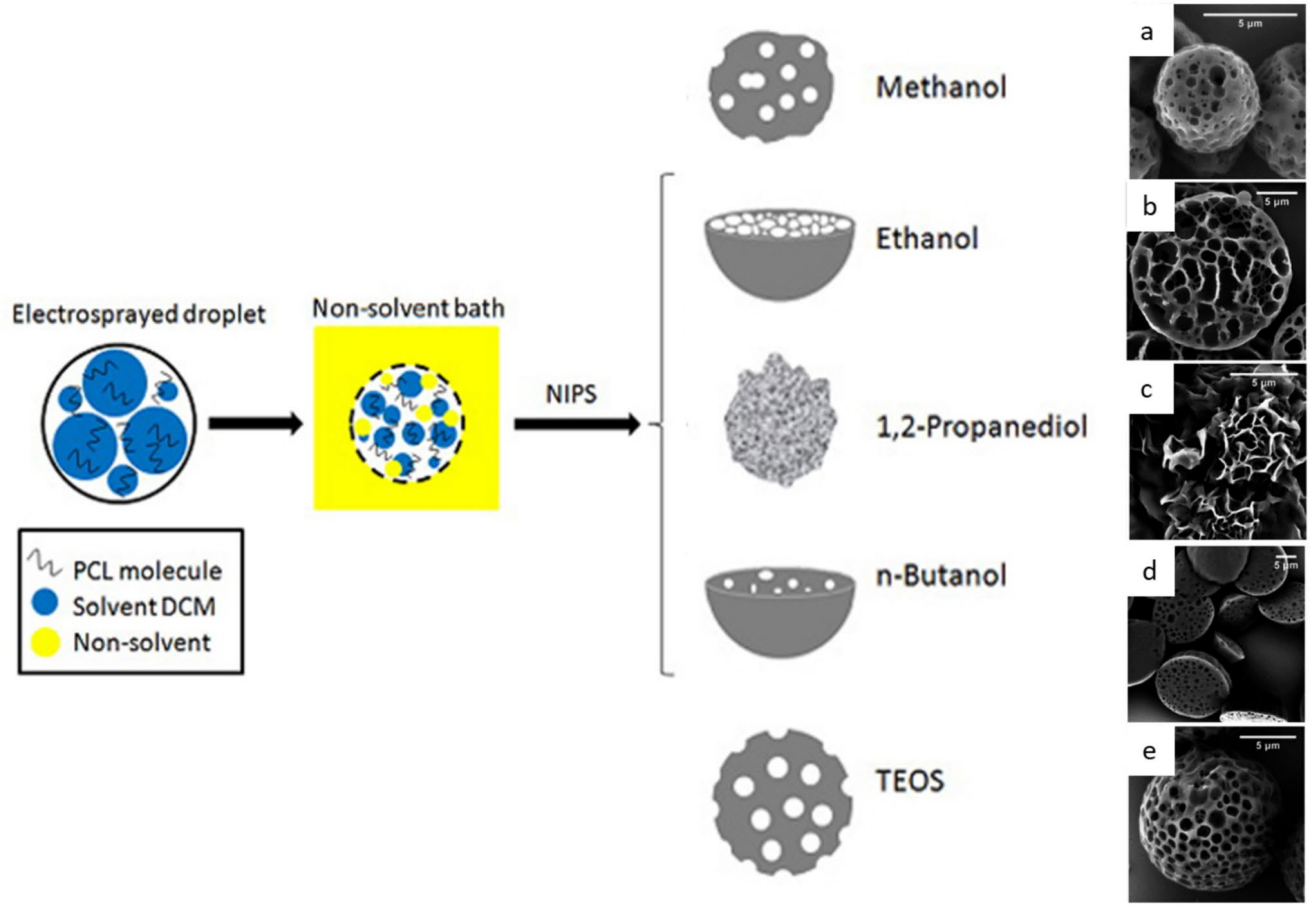
Preparation of porous PCL particles by electrospraying into a collection bath for non-solvent induced phase separation (NIPS). Scheme of preparation process and SEM pictures of obtained samples. Non-solvents: (**a**’) methanol, (**b**’) ethanol, (**c**’) 1,2-propanediol, (**d**’) n-butanol, (**e**’) tetraethyl orthosilicate. Adapted and reprinted from [50] with permission from MDPI under a Creative Commons Attribution 4.0 International License (https://creativecommons.org/licenses/by/4.0/).

Electrosprayed particles can also be collected in fluid baths to induce particle hardening by NIPS. As illustrated for PCL particles using ethanol, methanol, butanol, or tetraethyl orthosilicate as collection medium, the different hardening fluids led to strongly different pore morphologies (Fig. 2) [50]. Other interesting parameters include the polymer concentration and the additional usage of porogens.

The particle production by electrospraying process is limited to certain (typically low) polymer concentrations, while fibers instead of particles may be formed at higher polymer concentrations (electrospinning rather than electrospraying) [73, 74]. However, within the limited concentration range suitable for electrospraying, higher polymer concentrations were shown to produce particles with fewer and smaller surface pores [75]. In some cases, porogens were added to the polymer solution to promote pore formation by an additional mechanism [54, 59]; however, this does not appear to be a highly significant parameter in the fabrication of porous particles via electrospraying. Further effects, such as different electrospraying setups and other experimental parameters, have been reviewed in more detail recently [76, 77].

Different needle geometries can be particularly interesting for core–shell and Janus particles. An example are co-axial needle setups, which consist of a capillary feeding a solution of the core face that is placed inside another capillary carrying the outer (shell) solution. The flow rates (i.e., ratios) of both fluids can be independently tuned through the respective channels and eventually meet at the tip of the nozzle to produce core–shell particles [78–80]. In contrast, non-concentric needle setups with parallel ports seem to work best for producing Janus microparticles [81, 82]. In principle, these types of sub-structured particles may also be tuned to show distinct levels of porosity by the measures introduced above, such as varying solvent types [65] or collection media [83].

**Fig. 3:**
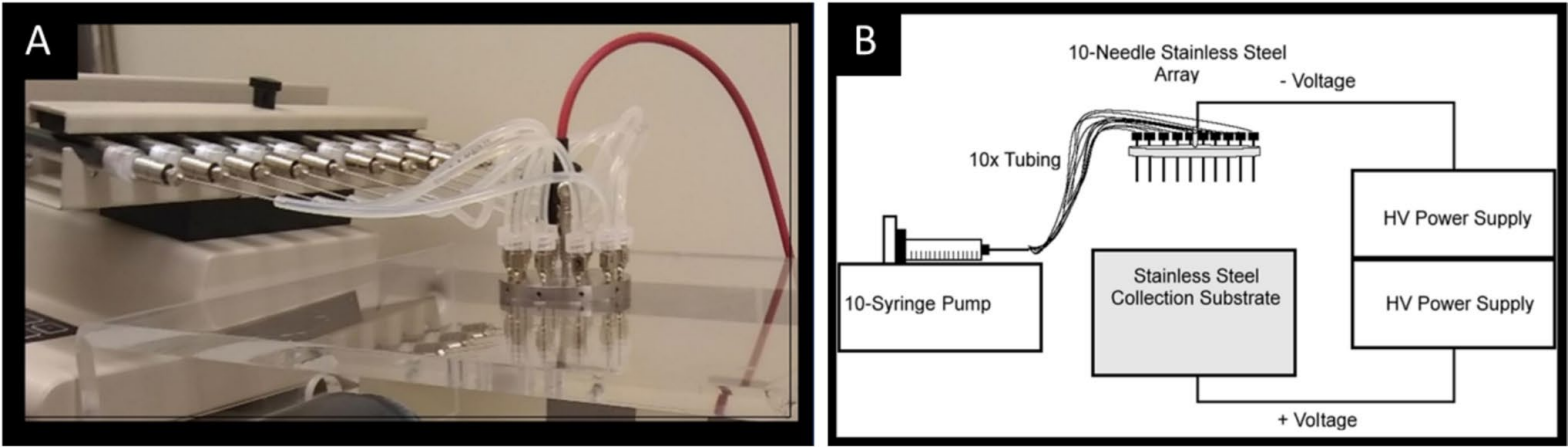
Enhanced throughput of electrospraying by parallelized multi-needle setups. (**A**) Photograph of a 10-needle array, attached to high voltage power supply. (**B**) Scheme of experimental setup. Adapted and reprinted from [86] with permission from Elsevier.

All the studies mentioned above utilize electrospraying in small-scale setups and were conducted primarily in academic research settings. Under these conditions, the productivity of electrospraying is considered very low (milligrams per hour), as it typically operates with single nozzles and requires diluted polymer solutions. Electrospraying can be rated as suitable for lab-scale manufacturing and particle engineering processes, as it produces homogenous particle size distributions and morphologies, and, in some cases, eliminates the need for additional drying steps. However, points of concern for pharmaceutical production processes include the limited throughput of this technique, coupled with the large volume of organic solvents required relative to the processed polymer mass (due to the use of diluted polymer solutions). Nevertheless, in recent years, there has been technological progress in the production scale of electrospraying and electrospinning devices, along with an increase in the number of suppliers for these machines [84]. Enlarged setups such as multiple nozzles running simultaneously have already been used to increase the throughput of particle production [85–88] (Fig. 3).

The productivity can also be increased by pressurized gas-assisted electrospraying (EAPG), a process combining the pneumatic spraying of a polymer solution with the principle of particle drying in a high-voltage field. This process apparently can lead to a higher mass throughput than conventional electrospraying set-ups (up to 1 kg/h) [89] and has also been applied to prepare particles for drug delivery [90, 91]. Other milestones on the path to industrial processing in the pharmaceutical sector include the development of the first pilot-scale processes, followed by GMP-certified and agency-approved manufacturing plants. Bioinicia from Spain, which was the first company that achieved this milestone for nanofibers (electrospinning) to the best of our knowledge, is also pushing boundaries for large-scale electrospraying by having opened a GMP-certified EAPG particle plant in 2023. Apparently, they use multi-needle setups (up to 5000 needles) for very high throughput and, if needed, also field deflectors to prevent jet interference. Other companies, such as Inovenso or Elmarco, also offer platforms and devices for pilot- and industrial-scale production of fibers and particles.

Overall, as the upscaling of the electrospraying processes for specific pharmaceutical use-cases is not reported in literature to date, it can be concluded that there are technological challenges and/or inefficiencies compared to competing production technologies. Although electrospraying offers flexibility in the manufacturing of different particle types, such as by using different needle setups [92], other established pharmaceutical production processes for particle engineering, such as spray-drying, are well known and offer much higher throughput rates. However, the sister technology of electrospraying, electrospinning, has long been facing a similar criticism but is now a step further ahead due to needleless set-ups, which already allow the upscaled production of fiber meshes for various applications at industrial scales [84, 93, 94]. The application of such up-scaled electrospinning processes has been reported in the pharmaceutical sector, such as by post-processing of electrospun fibers into other dosage forms like tablets [95, 96]. Considering the fact, that electrospinning and -spraying devices are very similar, with only slight differences in process and formulation parameters, it can be expected that technological advancements such as the construction of multi-needle/needleless setups that pushed the sister technology, electrospinning, may also promote the development of higher throughput for electrospraying.

### Droplet-based Microfluidics

Microfluidic techniques allow the handling of particularly small amounts of fluids while providing a precise control of flow dynamics, which may differ from conditions at the macroscopic scale [97]. Microfluidic devices (chips) typically include a network of microscale channels made of glass, silicon, or other polymeric materials. According to the principle of this method, droplets can be generated at the junctions of channels where two or more immiscible fluids are pumped together. The dispersion into the continuous phase is mediated – depending on the respective channel geometries, arrangements, and flow conditions – by principles such as lateral shearing, squeezing of threads by the surrounding media, or Laplace pressure gradients [98]. Under the well-defined flow conditions in microfluidic devices, highly reproducible events of droplet formation can be accomplished leading to monodispersity of particles [99].

Since microfluidic techniques enable fine manipulation of fluid flow, unique water-in-oil-in-water (w_1_/o/w_2_) double emulsions can be produced that have a controllable number of w_1_ water droplets dispersed in each droplet of the dispersed organic phase, which is impossible to realize by batch emulsification techniques. Microparticles with 1 to 4 bigger pores were prepared using such procedures, with the number of pores corresponding to the number of entrapped w_1_ water droplets [100–102]. As the entrapment process can be highly reproducible over long production times in stable microfluidic conditions, droplet-based microfluidics provides a level of control of particle ultrastructure that can hardly be reached by other techniques.

Beyond the number of encapsulated droplets, particle porosity can also be regulated by the composition of the respective phase, i.e., formulation parameters. More specifically, formulation parameters known from batch emulsification techniques (e.g. polymer types and concentrations, emulsifier concentration, additives, emulsion types) can be transferred to microfluidic systems. For instance, a decrease of the polymer concentrations in the dispersed phase and an adjustment of premixed w_1_/o ratios in double emulsions can affect the particle porosity [103, 104]. Furthermore, the outcome of the particle morphology depends strongly on the used matrix material and the applied process conditions [105].

The use of porogens, as introduced in Sect."Principles of Pore Creation and Pore Characteristics", is the most effective route to fabricate porous particles in microfluidic systems [106, 107]. Different porogens have been employed for preparing porous PLGA and PLA microspheres by microfluidics (Table I), including ammonium bicarbonate [31, 107], gelatin [22, 35, 103, 108–110], collagen [111], PBS buffer [22], camphene [112, 113], smaller porous silica particles [101], and assemblies of perfluorinated dendrimers or hyperbranched polymer skeletons [114, 115]. A tuning of open porous structures, including increased overall porosity and/or larger pore sizes, can be achieved by increasing the concentration of camphene as the porogen, which crystallizes upon solvent removal and is subsequently sublimated during freeze-drying to create pores [112]. When a gelatin solution was used as a porogen in PLGA particles that also contained fragments of decellularized extracellular matrix (dECM) in their w_1_ phase, it was observed that increasing gelatin concentrations, in combination with higher ultrasonic pre-mixing power during preparation of the w_1_/o emulsion, led to significantly larger pore diameters [116]. Interestingly, the pore sizes and overall particle sizes also depended on the content of dispersed dECM and increased in some but not all cases at the highest (50 wt.%) compared to the lowest (25 wt.%) dECM loading. A similar study fabricated porous PLGA particles and investigated the influence of polymer/concentration, porogen concentration (gelatin), and different w_1_/o ratios on the pore sizes [104]. Apparently, pore size tuning was achieved by increasing gelatin concentrations with additional inverse effects of the w_1_/o ratios on pore sizes. Another example of creating pores in a microfluidic production process was reported for dendrimeric or hyperbranched perfluorinated polymers, which can stabilize gas microbubbles in certain formulations (supramolecular complexes with a dye having a perfluorinated alkyl anchor) that are introduced in organic solutions of PLGA and act as templates for pores. Changes in the molecular weight and their molecular architecture (dendrimer, hyperbranched skeleton) allowed for the tuning of pore sizes, presumably by alteration of the microbubble sizes formed in the presence of the dye-dendrimer complex [114, 115, 117].

**Table I:** Examples of Porous Microparticles Prepared by Electrospraying, Microfluidics, and with Supercritical CO_2_

Polymer	Fabrication method	Porogen	Modified parameters	Particle sizes [µm]	Porosity [%]	Pore sizes [µm]	Reference
PCL	Electrospraying	n.a	Nonsolvents: Tetraethyl- orthosilicate, butanol, ethanol, methanol	11.70 ± 1.42 12.83 ± 2.53 20.35 ± 4.48 7.00 ± 1.18	n.d	0.49 ± 0.13 0.56 ± 0.33 1.35 ± 0.61 0.22 ± 0.18	[50]
PCL	Electrospraying	n.a	Solvent/Nonsolvent ratio (Ethanol:DCM)**↑**	6.14 ± 0.59	n.d	0.1–1.0	[53]
PMMA	Electrospraying	n.a	Humidity **↑**	n.d	n.d	0–0.22	[60]
Gelatin/ Chitosan	Electrospraying	n.a	G/C ratio **↑** Voltage **↑** Flow rates **↑**	250–500	83–93	n.d	[62]
PLA	Electrospraying	n.a	Humidity **↑**	n.d	24–56	n.d	[72]
PLGA PLA	Microfluidics	ABC	Flow rates of cont. phase **↑**	77–43 112–53	71–78 81–86	4–3 23–12	[31]
PLGA	Microfluidics	Gelatin	n.a	246.3 ± 17.7	n.d	48.3 ± 7.2	[108]
PLGA	Microfluidics	Gelatin	n.a	55.92 ± 10.11	n.d	11.31 ± 4.4	[110]
PLGA	Microfluidics	Gelatin	Mass ratio gelatin:PLGA **↑**	260–540	n.d	20–55	[109]
PCL	Microfluidics	Camphene	Flow rate ratio ↑	42–58	n.d	3–19	[113]
PCL	Microfluidics	Camphene	Camphene concentration ↑ Solidification temperature ↑	170.2–329.5 n.d	n.d	11.4–120.1 35–60	[112]
PLGA	Microfluidics	Gelatin	Gelatin concentration ↑	443–650	n.d	13.6–80.1	[116]
PLGA	Microfluidics	Gelatin	Gelatin concentration ↑ PLGA concentration ↑	269–397	n.d	8.4–31.1 8.4–32.7	[104]
PLGA	scCO_2_	n.a	Untreated Post-treatment	5.14 ± 2.8 12.96 ± 2.9	28.24 ± 1.9 83.52 ± 3.4	0.78 ± 0.05 1.76 ± 0.47	[150]
PLGA	scCO_2_	n.a	Untreated Post-treatment	2.2 ± 0.8 13.8 ± 1.3	39 ± 4.2 92.38 ± 2.96	0.09 ± 0.01 0.19 ± 0.03	[149]

*n.a.: not applicable*

*n.d.: not determined*

*ABC: ammonium bicarbonate*

Overall, when selecting a certain composition of the formulation, it should be considered that too high concentrations of porogens could result in dysmorphic and collapsed particles due to the mechanical instability of the remaining polymeric scaffolds as observed, e.g., when rising the Camphene concentration up to 80% [113]. In addition to the use of additives acting as porogens, pore formation in particles prepared by microfluidics will also proceed via osmotic effects of encapsulated drugs or the simple presence of an inner water phase in double emulsions [118–120].

**Fig. 4:**
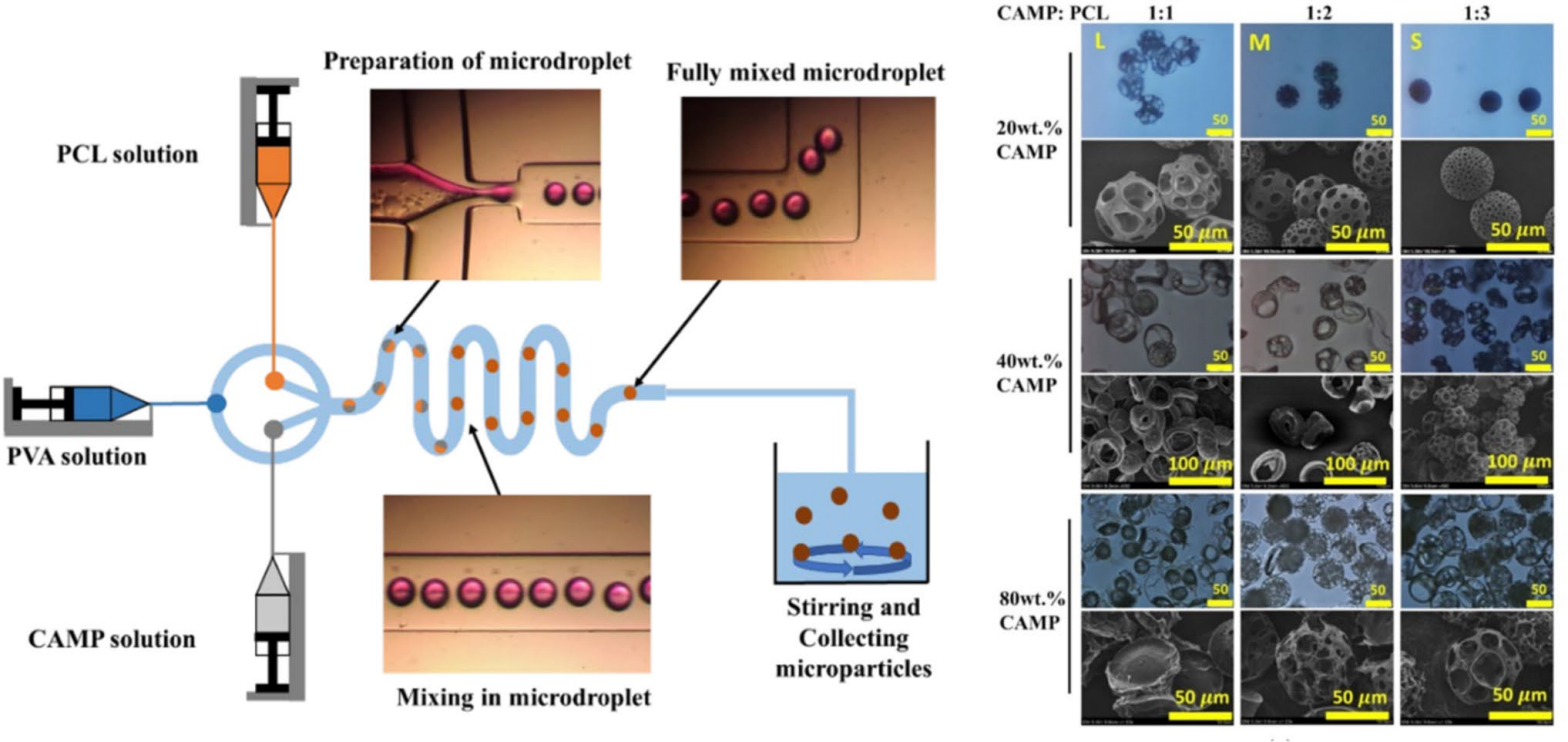
Microfluidic production of porous microparticles. (Left) Scheme of microfluidic setup and droplet formation. (Right) Images of particles depending on the flow rate ratios of the camphene (CAMP; varying wt.% in DCM) and PCL solutions (3 wt.% in DCM) forming a mixed dispersed phase. Additionally, the concentration of CAMP in the CAMP feeding solution was varied (20, 40, and 80 wt.%). Flow rates of the dispersed phases: 1:1 (0.5 mL/h CamP and 0.5 mL/h PCL); 1:2 (0.33 mL/h CamP and 0.67 mL/h PCL); 1:3 (0.25 mL/h CamP and 0.75 mL/h PCL). The flow rates of the continuous phase flow (2 wt.% PVA) were 5, 10, and 20 mL/h for 20, 40, and 80 wt.% CAMP. Adapted and reprinted from [113] with permission from MDPI under a Creative Commons Attribution 4.0 International License (https://creativecommons.org/licenses/by/4.0/).

A unique feature of microfluidics is that the combination of both the composition of the fluid phases and their feeding ratios can be used to control product properties. Additionally, channel geometries can be adjusted. While these parameters are primarily known to affect particle sizes [121–123], flow rate ratios in w/o/w procedures can also alter particle morphology [124]. If the porogen is added to a distinct phase, the particle porosity can be modulated by fine-tuning the flow rate ratios of the different phases [31, 113, 125]. Increasing flow ratios towards the polymer phase at a constant porogen concentration (in this case 20% camphene) led to a controllable decrease of both the sizes of PCL particles as well as the pore sizes (Fig. 4) [113]. For improved control of flow ratios and, thus, of particle morphology, separated microfluidic chips have been used in some cases for the primary (w_1_/o) and secondary ([w_1_/o]/w_2_) emulsification steps [125].

The spectrum of formulation parameters also includes solvents/non-solvents. When different types of solvents were compared as solvent for the o-phase, dichloromethane and chloroform were observed to be advantageous in terms of producing a spherical morphology and porous ultrastructure of PLGA particles compared to dimethylcarbonate and ethylacetate, which are solvents with a higher miscibility with water [22]. A higher water miscibility of o-phase solvents typically causes a faster mass transport of solvent into the continuous phase and thus a faster polymer precipitation at the interface. This rapid solidification of the matrix polymer may lead to the formation of core–shell particles that could collapse when remaining solvent escapes from the core [126]. Dichloromethane seems superior due to its low water miscibility, though a small amount of water May diffuse into the nascent particles creating porous structures. Phase separation induced by combinations of solvents and non-solvents added to the polymer phase for electrospraying, as discussed above, has also been employed in droplet-based microfluidics, for instance, by using 2-methylpentane as a non-solvent additive for PLA or PLGA [127].

While the majority of porous polyester microparticles processed by microfluidics had an open porous structure [22, 31, 108, 109, 112, 113, 125], which is particularly interesting for pulmonary drug delivery [128, 129] and tissue engineering [130, 131], some other studies showed particles with a dense and smooth surface but high core porosity [107]. Interestingly, also Janus particles with a one-sided porosity at the particle surface could be produced by phase separation of collagen (w_1_ phase) and PLGA (o phase) due to w_1_ phase coalescence during particle hardening [111].

**Fig. 5:**
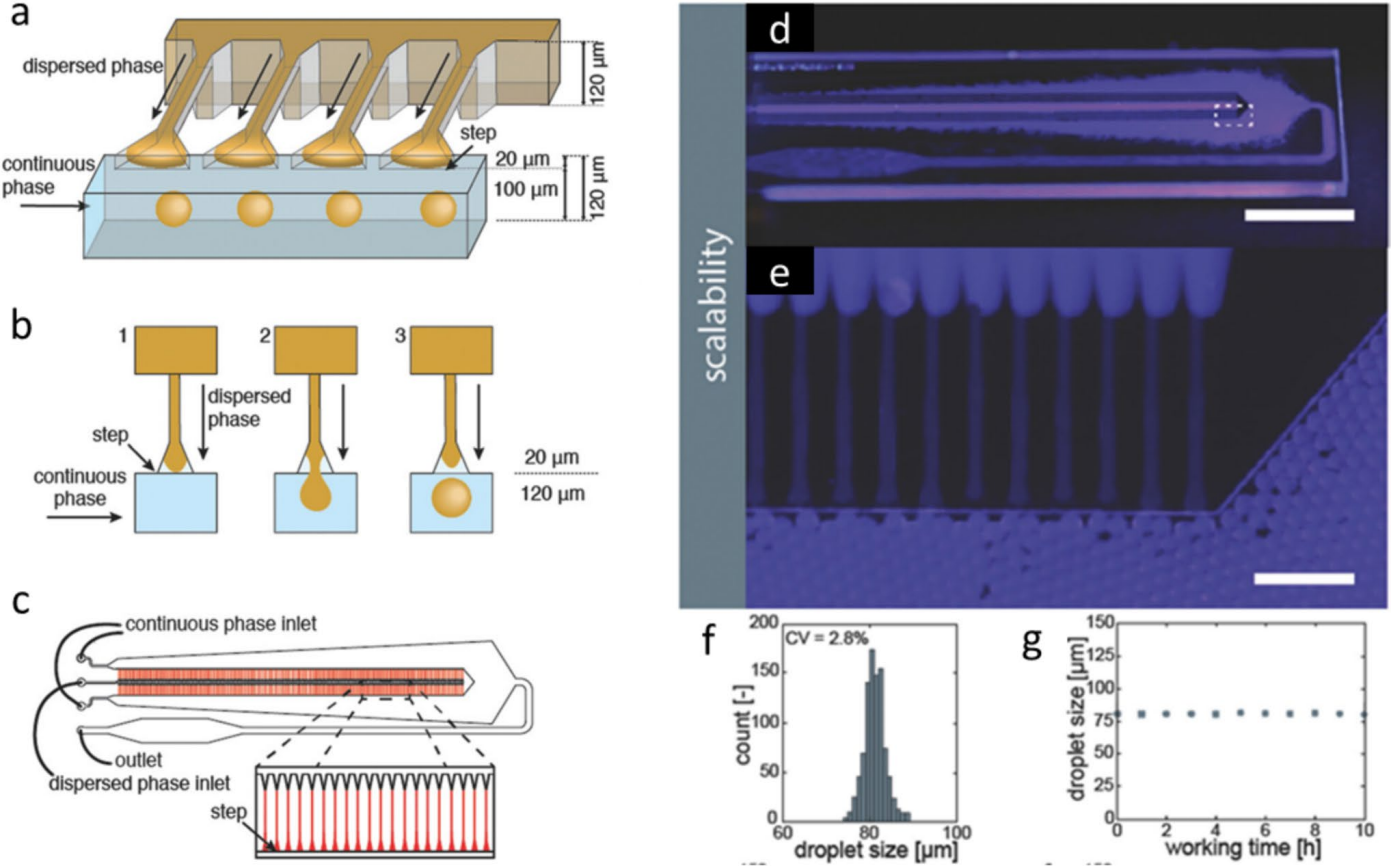
Upscaling of microfluidic particle production by numbering-up of droplet forming units in a step emulsification device. (**A**) Schematic of a step‐emulsification channel arranged with parallelized droplet makers. (**B**) Illustration of droplet formation (**C**) Layout of a microfluidic emulsification chip with 364 droplet forming unit. (D-E) Photographs of the microfluidic glass chip during operation (Scale bars: (**D**) 1 cm, (**E**) 500 µm). (**F**) Size distribution of hexadecane‐in‐water droplets with a mean size of 80.9 μm and a coefficient of variation (CV) of 2.8%. (**G**) Droplet size homogeneity during Long term operation as measured over 10 h. Adapted and reprinted from [135] with permission from Wiley.

In an overall assessment, droplet-based microfluidics typically provide much better results in terms of particle size distributions up to perfect monodispersity compared to conventional batch emulsification techniques. In some cases, higher encapsulation efficiency by microfluidics compared to batch fabrication was reported for hydrophilic drugs like metformin hydrochloride [132]. Additionally, given the very standardized flow conditions and droplet formation events of one drop after another, less batch-to-batch variations can be expected [133]. At the same time, due to the consecutive droplet formation, microfluidic procedures are much more time-consuming compared to the simultaneous droplet formation in batch emulsification methods. However, the productivity of microfluidic techniques can be substantially increased by numbering-up (parallelization rather than conventional scale-up). This means, on the one hand, operating multiple chips simultaneously, which, however, also requires multiple sets of essential infrastructure, such as pump systems. Therefore, on the other hand, setups have been developed that incorporate multiple sets of channel junctions (droplet-forming units) arranged in defined geometries, ensuring identical flow conditions at each unit and allowing operation with a single set of pumps [98, 134, 135] (Fig. 5). Additionally, there are several approaches that demonstrated an increased productivity per channel without detrimental effects on particle monodispersity. This includes an alteration of the time point/position of surfactant addition for droplet stabilization [136] or the operation of capillary microfluidic devices under tip-streaming rather than dripping conditions [123].

Importantly, the larger scale production with parallel operation of chips/droplet-forming units has to be subjected to continuous supervision. In particular, flow dynamics in multi-channel devices may severely change upon clogging of individual channels, thus affecting the particles produced at the other droplet-forming units. Therefore, in contrast to batch emulsification methods by conventional mixers, microfluidic production processes require perfectly well dissolved materials in the different phases, particularly in the organic polymer phase (no lumps or gel-like particles), which otherwise could cause inhomogeneous streams inside the channels.

Other important aspects of upscaling microfluidic devices include the types of materials and the methods used to fabricate the chips. Several materials like PDMS, glass, steel, and thermoplastic polymers as well as different chip fabrication methods (e.g. photolithography, hot embossing, injection molding, 3D printing) may be considered, as reviewed in detail by other authors [137, 138]. Obviously, it is important to employ materials in chip fabrication that are compatible with the reagents (solvents) used during particle production and neither dissolve nor swell, which can be critical with some organic solvents. Additionally, particularly when thinking of 3D printing of chips, the resolution of the printing technique should be critically evaluated in terms of both smoothness (flow perturbations) and tightness (device leakage) of the channels. To the best of our knowledge, industrial-scale droplet-based microfluidic systems have not been used in the commercial production of drug carriers yet, but efforts are being made to bridge the gap between small-scale and industrial-scale production. Companies like Microcaps (Switzerland) or Emultech (Netherlands) are developing large-scale solutions for industrial applications. For instance, Microcaps is using an alternative setup with stacked chips, which includes multiple channels running simultaneously and a collection of nascent particles in a bigger reservoir that can facilitate larger scales.

Other techniques that allow an enhanced throughput while enabling narrow particle size distributions are based on membrane emulsification, specifically crossflow membrane emulsification, where droplets are typically formed by shear fgenerated by the stirred continuous phase in the collection chamber [139]. For instance, SPG Technologies (Japan) offers special cylindrical glass membranes, which can be incorporated into semi-continuous lab-scale devices [140–142] and are claimed to be compatible with large-scale production equipment. Another company, Micropore (UK), uses membranes and devices made of steel.

In contrast to droplet-based microfluidics, nanoprecipitation within flow-driven systems has enabled the large-scale and GMP-certified manufacturing of lipid nanoparticle vaccines, such as those used for COVID-19 [143]. It should be mentioned that the nanoprecipitation in this case was based on turbulent-flow jet mixing, during which solvent exchange takes place, ultimately leading to spontaneous (i.e., less controlled, statistically distributed) particle formation processes.

Given the fact that flow-driven techniques with micromixing equipment (specifically nanoprecipitation) have already advanced into pharmaceutical manufacturing of drug products, droplet-based microfluidics may likewise find practical applications in industrial-scale particle production. This is particularly expected when challenges of conventional batch methods – such as variations in particle sizes, morphology, and drug loading [144] – are overcome, the required investment is low (using pumps and reusable microfluidic devices) compared to large scale machinery, and the throughput meets market demands such as for niche therapeutic applications.

### Treatment with Supercritical Fluids

Supercritical fluids (SCF) are obtained at a temperature and pressure above the critical point of a given substance, where distinct liquid and gas phases no longer exist. In this state, SCF exhibit unique properties that are intermediate between those of gases and liquids such as moderate density, low viscosity, and high diffusivity. The most commonly used supercritical fluid is carbon dioxide (CO_2_) due to its critical point at relatively low temperature and pressure (≈ 31°C/74 bar), low-cost, and non-toxic nature.

SCF-based processing is often applied to prepare porous macroscopic scaffolds under solvent-free conditions [145] or to enhance the matrix porosity and pore sizes of initially non- or low porosity systems [146]. The underlying principle is that a SCF such as scCO_2_, once absorbed into a material, transitions towards the gaseous state upon depressurization, leading to the formation of CO_2_ gas bubbles within the material. This results in the expansion (foaming) of the matrix material.

The application of SCF techniques to prepare porous PLGA or PLA particles is less common due to the need for specialized equipment, which is not widely available in pharmaceutical research [147]. However, there are examples where particles prepared using conventional batch emulsion techniques followed by freeze drying were subsequently subjected to post-treatment with scCO_2_ [148, 149]. This treatment enabled an increase in particle porosity as desired. Additionally, as SCFs have good solvent power for many substances, a beneficial reduction of the residual DCM content was observed after SCF treatment for particles initially formed by using DCM. Interestingly, at the same time, this procedure led to a modification of the drug release profiles, e.g., of risperidone towards a more desirable linear pattern [149, 150].

The mandatory depressurization step during SCF processing can have varying effects on different materials. When comparing PLA and different PLGA grades after treatment with scCO_2_, PLGA particles showed an increase in size, along with the formation of pores, while the morphology of PLA particles remained unaltered [149]. The authors suggested that the crystallinity of PLA significantly limits the mobility of the polymer chains and the absorption of scCO_2_, which are essential for scaffold expansion by this method.

The application of SCF can also go beyond pore formation/foaming. It was shown that the joint exposure of bevacizumab-coated PLA nanoparticles and PLGA microparticles to scCO_2_ resulted in a transportation (loading) of the PLA nanoparticles into the pores of the expanding – and thus now porous – PLGA microparticles [151].

**Fig. 6:**
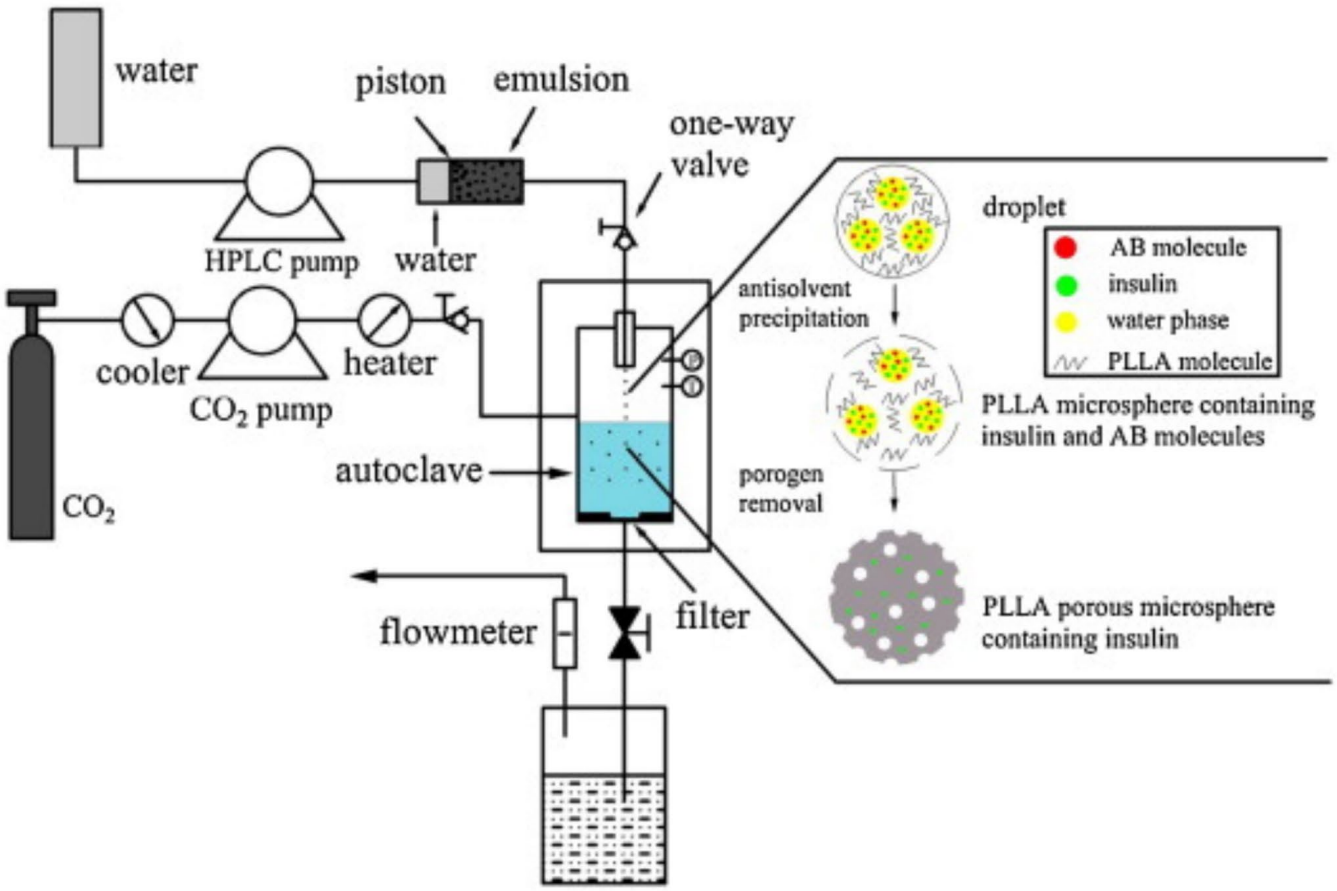
Schematic diagram of the preparation process of porous PLA particles with sub-critical compressed CO_2_. The first emulsion (w_1_/o) is pumped through a nozzle and into a steady atmosphere of compressed CO_2_ in an autoclave, from which porous particles can be collected after depressurization. Reprinted from [128] with permission from Elsevier.

Another process that involves compressed CO_2_ for particle preparation comprises the hydraulic dispersion of a polymer solution into droplets/particles, followed by particle hardening and solvent extraction via precipitation with compressed antisolvent (PCA process), and foaming – all carried out in a high-pressure spray and extraction reactor in a single procedure (Fig. 6). In some cases, ABC was added as an additional porogen [152, 153]. This use of porogens is reasonable when the system is operated in the sub-critical regime (highly compressed gas but staying below the supercritical conditions), i.e., at conditions that still present phase boundaries between the compressed gas and the polymer solution, which are necessary for particle formation. Under subcritical conditions, the level of achievable porosity and the extent of open pores at the particle surface are often limited without an additional porogen [154, 155]. The ABC-assisted particle production via PCA allowed for the fabrication of irregularly-shaped PLA particles loaded with insulin as an active ingredient. Importantly, by combining different pore-forming principles, a sufficient porosity could be achieved, with aerodynamic diameters matching the needs of pulmonary delivery [152, 156].

In general, the scale-up of supercritical fluid-based processes is well established, e.g., in food technology [157]. Often, the solvent or anti-solvent properties of SCF are utilized there. Given the solubilization power of scCO_2_ for many (not too polar) active pharmaceutical compounds (API) [158, 159], it will be important to ensure that the scCO_2_-based foaming processes will not result in the extraction and loss of API when applying this process in the pharmaceutical sector. Despite not using polymers, it is interesting to note that a scaled-up semi-continuous production process of quercetin-loaded micelles was operated through scCO_2_-based extraction of organic solvent from o/w emulsions, which could potentially be applied to microparticle production as well [160]. Although processes using supercritical fluids are less common than established methods like spray drying, this technique shows some potential to be considered for particle engineering processes. One aspect – although potentially more relevant for the foaming of larger objects – is the improved ecological balance of production, as high-pressure polymer processing with SCF may enable a lower energy consumption and solvent usage [161].

## Characterization of Porosity

The analysis of overall porosity, pore size distributions, and/or pore geometries is crucial for understanding the properties of microparticles and their applications. According to the IUPAC, pores are classified by size as micropores (< 2 nm), mesopores (2-50 nm), and macropores (> 50 nm) [162]. As most literature on porous microparticles for pharmaceutical applications reports macroporous characteristics, with voids up to several micrometers, this section briefly summarizes suitable characterization methods for macropores. More comprehensive discussions can be found in specialized reviews on this topic [163, 164].

Easily accessible optical methods, such as light microscopy, are powerful tools for analyzing particle sizes in aqueous dispersions or, after proper sample preparation, in the dry state. However, light microscopy is less frequently used to determine pore sizes, as the pores of pharmaceutically relevant polymer microparticles (mean particle sizes of injectable depot formulation most often in the range of 5–100 µm) are typically not visible in wet dispersion (unless dye-based visualization by confocal microscopy can be employed for relatively large particles/pores). Additionally, limitations of light optics (e.g., resolution, material-dependent opacity, or the curved surface of particles complicating focus plane detection) present further challenges for wet- and dry-state analysis.

Electron microscopy, particularly scanning electron microscopy (SEM), is the gold standard for visualizing porous particles in the dry state, given its high resolution and suitability for imaging three-dimensional objects. SEM can provide direct insights into surface porosity by measuring pore sizes in images using external software (e.g., ImageJ). Challenges associated with SEM include the limited thermal stability of polymers, like PLGA, under the electron beam, which can cause pores and particles to deform in case of improper instrument settings and charging of the samples. To assess internal porosity, experienced operators are needed, given the complex and time-consuming procedures for sample preparation by (cryo)-ultramicrotomy [12]. In cases where microtomy instruments are not available and/or when a lower quality of cross sections is acceptable, cutting with a razor blade may also be used with reasonable results [8, 165, 166], but should involve a critical assessment, as particle deformation and smearing of material over the porous structure can occur. Combinations of SEM with additional techniques, in particular focused ion beam (FIB)-based *in situ* cutting of single particles within the SEM, enable very detailed insights into the particle ultrastructure via multiple cross-sectional views [167]. Importantly, microscopic techniques only provide information on small quantities of sample material, placing significant responsibility on operators to select and provide representative images for subsequent qualitative or semi-quantitative analysis.

Quantitative information on batch porosity and pore size distributions can be obtained by methods like gas adsorption or mercury intrusion porosimetry. Among these, mercury intrusion porosimetry is the best-known and most widely applied method for porous drug carriers due to its ability to cover a wide range of pore sizes (10 nm – 300 µm) and to provide comprehensive insights not only into overall porosity and average pore sizes, but also into pore size distributions including the smallest and largest pore diameters of open pores [10, 168, 169]. However, this method requires larger sample quantities a compared to microscopy techniques, and handling of elementary mercury poses significant health risks, in addition to generating contaminated samples that require special waste containment.

In exploratory research, alternative characterization methods have been discussed to determine pore structures. Examples include micro-computed tomography (µCT) or nanoCT as non-destructive techniques for analyzing samples typically in the dry state. These methods generate 2D images of different sample planes, which can be combined to construct a three-dimensional model of the sample. For instance, the imaging of porous microstructures inside PLGA particles and the reconstruction of the porous network of risperidone-loaded PLGA particles have been reported using µCT or nanoCT [7, 8]. Challenges of µCT include resolution limitations (a few micrometers), while nanoCT provides higher resolution but is restricted to very small samples (usually single particles) and long measurement times, i.e., low sample throughput.

Another explorative method for determining particle porosity is based on sedimentation velocity, which depends on density differences between particles and the suspension medium and thus is affected by porosity (in addition to particle sizes). It was shown for PLGA particles that sedimentation analysis combined with Camera inspection of 50–100 particles can provide a good correlation with data from mercury intrusion porosity [170].

Overall, the most effective method for determining the pore size range and porosity of porous particles is a combination of techniques, most commonly microscopy together with mercury intrusion porosimetry.

## Conclusions

The platform of fabrication techniques for porous drug carriers is subject to ongoing technological advancements. Besides conventional batch emulsion techniques, methods like electrospraying, microfluidics, or treatment with supercritical fluids pose some interesting features that can be advantageous to reproducibly fabricate porous drug-loaded particles. Some formulation aspects of batch techniques and mechanisms of pore formation by adding porogens can be transferred and applied to those continuous methods. Despite existing technological challenges and process-related limitations of throughput, a scale-up of the here discussed alternative methods may be possible in principle, in some cases even being evidenced for other application fields or material systems. Still, as of now, these methods may be primarily suited for the cost-effective production of porous particles for niche applications that require high flexibility in production technology, rather than for the manufacturing of large-scale (ton-level) quantities.

## Data Availability

All data generated or analysed during this study are included in this published article.
